# Experiences and challenges of sexual and gender minority patients undergoing breast cancer treatment

**DOI:** 10.1016/j.amjsurg.2026.116947

**Published:** 2026-03-27

**Authors:** Gabi Barmettler, Sara Kohlbeck, Chandler S. Cortina, Olga Kantor

**Affiliations:** a Division of Breast Surgery, Department of Surgery, Brigham and Women’s Hospital, Boston, MA, USA; b Breast Oncology Program, Dana-Farber Brigham Cancer Center, Boston, MA, USA; c Comprehensive Injury Center, Medical College Wisconsin, Milwaukee, WI, USA; d Department of Psychiatry and Behavioral Medicine, Medical College of Wisconsin, Milwaukee, WI, USA; e Division of Surgical Oncology, Department of Surgery, Medical College of Wisconsin, Milwaukee, WI, USA; f Medical College of Wisconsin Cancer Center, Milwaukee, WI, USA; g Current affiliation: Nuvance Health Dyson Cancer Center, Northwell Health, Poughkeepsie, NY.; h C.S. Cortina and O. Kantor contributed equally and share senior authorship.

**Keywords:** Breast cancer, Treatment, Sexual orientation, Gender minority

## Abstract

**Background:**

This survey study sought to characterize the experiences and treatments of sexual and gender minority (SGM) patients with breast cancer.

**Methods:**

The WhySurg survey was modified to focus on SGM patients and distributed via social media, support groups, and clinic flyers between 2023 and 2024. SGM individuals, age ≥18, and a history of breast cancer were eligible. Descriptive and reflexive thematic analyses were performed.

**Results:**

50 individuals responded, with 31 (62%) completing it. Most (77%) were cisgender women, 16% nonbinary, and 3% transgender man. Most (58%) were lesbian/gay/homosexual, while 23% were bisexual, 13% pansexual, 13% queer, 3% asexual, 1 heterosexual, and 1 other. 29% experienced discrimination related to their SGM identity during treatment. Three themes emerged: 1) SGM identity influenced surgical choice, 2) respondents were reluctant to share SGM identity, and 3) appropriate surgical expectations are key to optimize gender-affirming patient-centered outcomes.

**Conclusion:**

SGM data is essential to ensure patient-centered surgical decision-making.

## 1. Introduction

The experience and treatment choices for breast cancer in sexual and gender minority (SGM) populations have been underrepresented in breast cancer research. SGM patients encompass a heterogeneous and diverse group of individuals who identify as lesbian, gay, bisexual, pansexual, transgender, nonbinary, intersex, asexual, queer, and/or gender diverse. Population-based survey data have found an increasing proportion of the population identifies as a SGM and recent data suggested 9.3% of the total United States adult population identifies as SGM in 2025,^1^ indicating the significance of understanding how breast cancer impacts this unique patient population.

Notable breast cancer disparities have been identified in the SGM population including increased breast cancer risk factors, such as increased use of alcohol and tobacco, inferior breast cancer screening rates, delays to breast cancer treatment, and inferior survival outcomes compared to cisgender heterosexual individuals ^2–6^. Breast cancer screening rates are notably lower amongst SGM populations, which is an essential tool to facilitate prompt diagnosis and early treatment to improve outcomes^7^ and while multifactorial, negative interactions with healthcare systems and providers contributes to decreased screening rates in this patient population.^8^ Additionally, intersectional stigma adds to delays in breast cancer care as SGM Black women have 5-fold increased odds of care delay in comparison to white heterosexual women,^8^ thus compounding disparate outcomes.

Despite these data, there is presently limited granular data on how SGM patients with breast cancer perceive their treatment options and their treatment experience with the intersection of their gender identity and/or sexual orientation and a breast cancer diagnosis. We aimed to better understand the intersection of SGM identity and breast cancer treatment among SGM patients with breast cancer.

## 2. Methods

This survey study was performed from 2023 to 2024 and distributed using the secure Qualtrics platform through Brigham and Women’s Hospital. SGM individuals aged ≥18 years who were previously diagnosed and treated for stage 0–IV breast cancer were eligible to participate. The survey was based on a modified version of the WhySurg study^9^ to have a breast cancer focus with questions related to SGM identity and breast cancer treatment (Appendix 1). This voluntary and anonymous survey was circulated through social media platforms, breast cancer support groups, and clinic flyers at two academic medical centers (Dana-Farber Cancer Institute and the Clinical Cancer Center at Froedtert and the Medical College of Wisconsin). Participants were able to elect to participate in this digital survey via a QR code or embedded link to the Qualtrics survey platform. This study was approved by the Dana-Farber Cancer Institute Institutional Review Board prior to survey distribution.

Descriptive statistics of the cohort were used to summarize respondents’ demographic characteristics, tumor variables, and treatment course for the overall cohort and by sexual orientation and gender identity. There were five optional free-text qualitative survey questions focusing on treatment decisions, provider interactions, and hospital systems. Optional free-text survey questions were analyzed using the reflexive thematic analysis approach using MAXQDA software. Responses were coded and refined by the four investigators to develop the final codebook. Themes were then inductively identified by each individual investigator which was then reviewed by all four investigators for final theme development. Researchers include 3 fellowship-trained breast surgical oncologists and 1 researcher (PhD), 2 of whom identify as a sexual minority, 1 as nonbinary, 2 cisgender women and 1 cisgender man). Data reporting follows the Consolidated Criteria for Reporting Qualitative Research (COREQ) checklist for qualitative data analysis reporting (Appendix 2).

## 3. Results

### 3.1. Cohort description

**Fig. 1.**
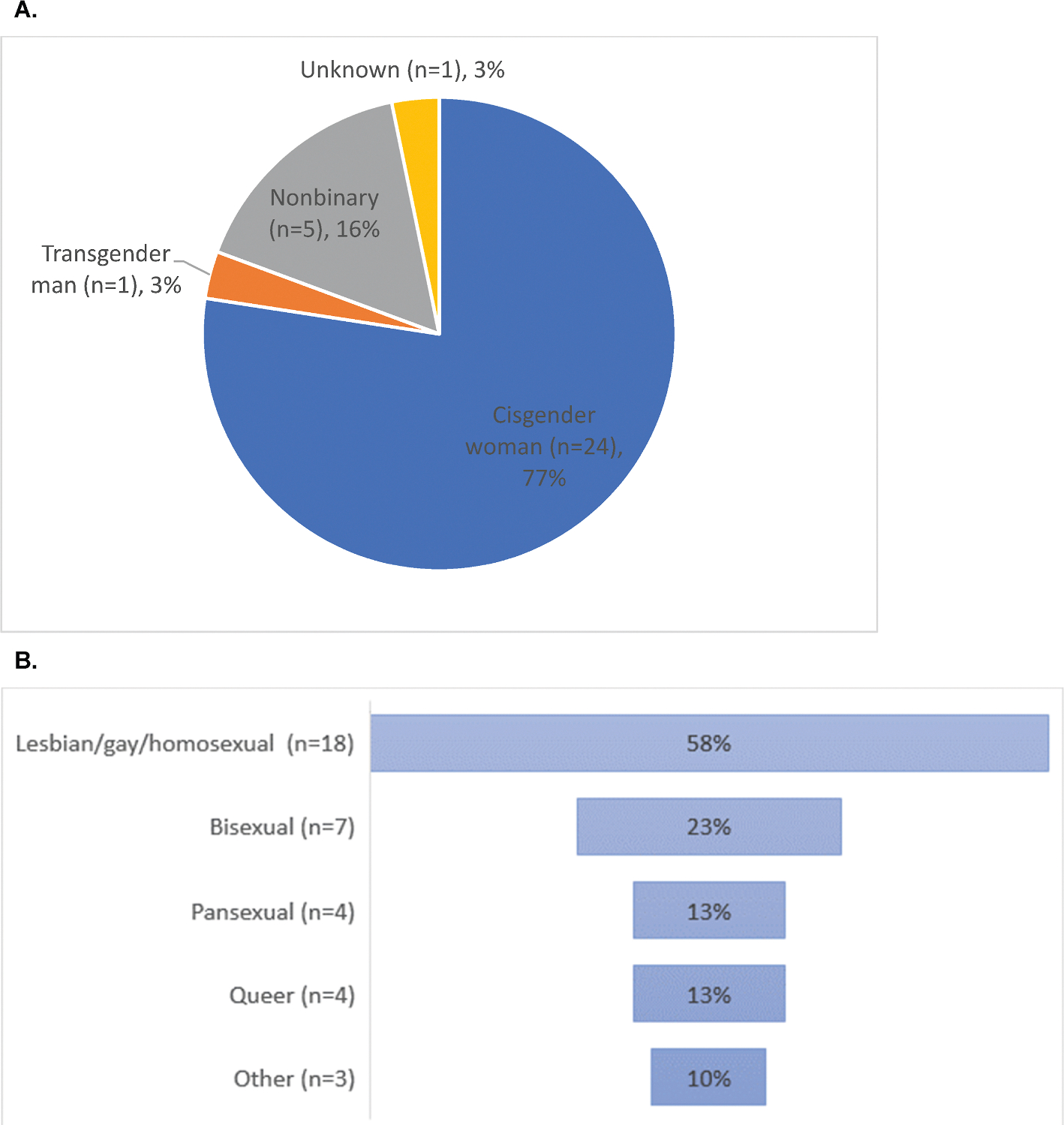
(A) Gender identity of respondents with breast cancer (N = 31), and (B) sexual orientation distribution within sexual and gender minority (SGM) respondents with breast cancer (N = 31).

**Fig. 2.**
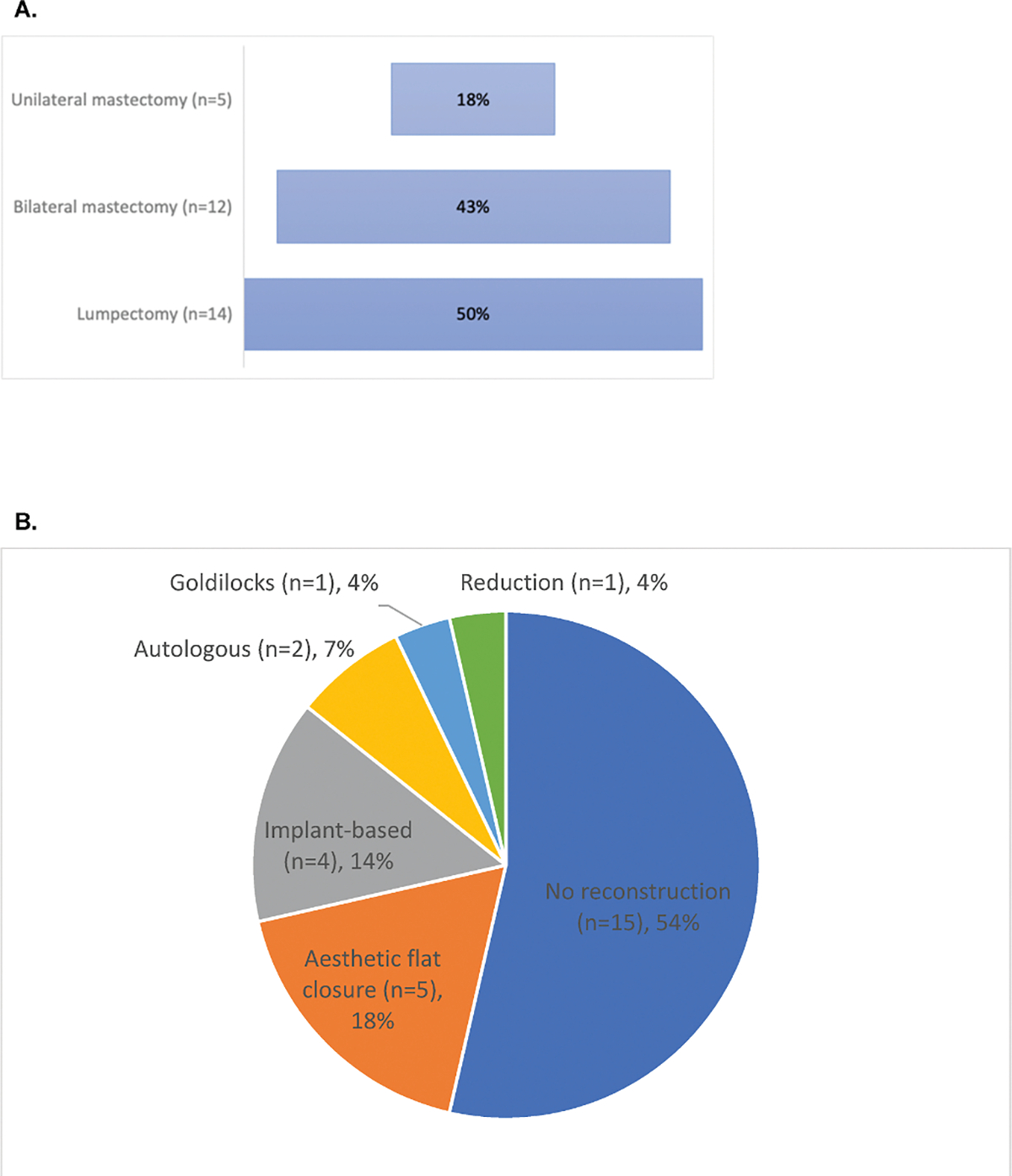
(A) Breast surgical management distribution in sexual and gender minority respondents with breast cancer (N = 28), and (2) breast surgical reconstruction distribution in sexual and gender minority respondents with breast cancer (n = 28).

A total of 50 individuals took the survey, with 31 (62%) completing at least part of the survey (Table 1). In terms of gender identity, the majority (n = 24, 77%) identified as cisgender women, 16% (n = 5) were nonbinary, 1 (3%) a transgender man, and 1 (3%) unknown (Fig. 1a). In terms of sexual orientation, most respondents (n = 18, 58%) identified as lesbian/gay/homosexual, 23% (n = 7) as bisexual, 13% (n = 4) queer, 13% (n = 4) pansexual, 3% (n = 1) asexual, 3% (n = 1) heterosexual, and 3% (n = 1) ‘other’ (multiple responses allowed) (Fig. 1b). Nearly half of respondents (n = 14, 45%) were between the ages of 36–50 at the time of their cancer diagnosis and most (n = 27, 87%) had stage I-III invasive breast cancer. Breast cancers were detected by screening mammography in 48% (n = 15), by clinical and/or self examination in 45% (n = 14), and 13% (n = 4) reported they were symptomatic (multiple responses allowed). In terms of treatment, 48% (n = 15) of respondents reported radiation therapy, 45% (n = 14) reported endocrine therapy, and 29% (n = 9) reported systemic chemotherapy. Twenty-four (77%) respondents reported genetic testing, of which 4 (17%) had a pathogenic germline variant. Ninety percent (n = 28) of respondents reported undergoing surgery: 14 (50%) with lumpectomy, 12 (43%) with bilateral mastectomy and 5 (18%) with unilateral mastectomy (multiple responses allowed) (Fig. 2a). Of the 13 (46%) respondents who reported reconstructive surgery, 5 had flat aesthetic closure, 4 underwent implant-based reconstruction, 2 autologous reconstruction, 1 had a goldilocks procedure, and 1 underwent oncoplastic reduction (Fig. 2b).

### 3.2. Qualitative analysis

The five open-ended questions ultimately resulted in three themes after undergoing comprehensive coding of patient responses to open-ended qualitative questions (Table 2).

#### Theme 1:

Sexual orientation and gender identity influences the decision for breast surgery in patients with breast cancer assigned female sex at birth.

Nearly half of those who had surgery (46%, n = 13/28) reported that their sexual orientation and/or gender identity directly impacted their decision for type of breast surgery. This was most notable for patients who identified as non-cisgender who had previously considered undergoing gender-affirming chest masculinization surgery prior to a breast cancer diagnosis. Similarly, patients who identified as bisexual reported that, because of their sexuality, their perception of options for physical body types was broader and it supported their decision to have an aesthetic flat closure after undergoing mastectomy and therefore omitting post-mastectomy breast reconstruction.

#### Theme 2:

Concerns regarding provider and healthcare system’s perception of an individual sexual orientation and gender identity influences patient comfort with disclosing sexual orientation and gender identity (SOGI) data to providers.

Twelve (39%) respondents disclosed their sexual orientation and/or gender identity to their surgeon. Reasons for not disclosing sexual orientation and/or gender identity for the remaining respondents included not being asked (n = 8, 26%), not believing it would factor into breast cancer treatment (n = 3, 10%), and fear of discrimination or marginalization (n = 2, 6%). Overall, 29% (n = 9) of respondents reported they experienced discrimination and/or marginalization related to their sexual orientation and/or gender identity during their treatment for breast cancer including misgendering in reception areas, judgement from surgeon regarding surgical choice and requiring psychiatry evaluation because of surgical decision for flat mastectomy.

#### Theme 3

Comprehensive pre- and post-operative setting of expectations by surgical teams with patients can aid in ensuring receipt of patient-centered surgical care that optimizes breast outcomes and embraces gender identity.

Most patients (n = 20, 65%) reported that their decision for surgery (lumpectomy vs mastectomy) was largely driven by surgeons, while only 3 patients reported they participated in shared decision-making with their surgeon team, and 2 patients reported they drove their decision (one for a genetic mutation and one who wanted bilateral mastectomies for gender dysphoria). After surgery, cosmetic outcome was notable in 11 patients (35%) who reported negative aesthetics and mental health. Notably, one patient who underwent mastectomy without reconstruction noted that the “concaveness is jarring” while another patient who underwent lumpectomy reported, “[my breasts] give me the feeling that I am perceived as a woman,” suggesting that improved pre-operative counseling may have resulted in a different surgical decision by the patient.

## 4. Discussion

In this survey study regarding experiences of SGM patients undergoing breast cancer treatment, an individual’s sexual orientation and/or gender identity influenced surgical decision-making, impacted how patients interacted with healthcare teams and systems, and influenced patient-reported surgical outcomes. Some respondents reported discrimination, misgendering, and assumptions regarding their identity during the treatment process. These data provide insight into opportunities to improve breast cancer care for SGM individuals.

Survey respondents identified that their sexual orientation and/or gender identity often influenced their decision to undergo mastectomy and forgo post-mastectomy breast reconstruction – opting to ‘go flat’. Options for flat aesthetic closure continue to expand and should routinely be discussed when counseling patients about their surgical options. Respondents mentioned the jarring feeling of “concavity” after flat closure and novel surgical techniques such as the Goldilocks procedure, in which the inferior pole dermal cutaneous flap of the breast is deepithelialized and used to create a low profile breast mound, could be used for select patients to minimize a concavity deformity.^10,11^ A dual oncologic mastectomy along with gender-affirming chest masculinization surgery (GACMS) should be discussed with patients who have a breast cancer and express interest in a masculine appearing chest.^12,13^ Patient-reported outcomes after GACMS demonstrate improved quality of life measures in nonbinary and transmasculine patients.^14^ The demonstrated improvement in quality of life after chest masculinizing surgery is contrasted in comparison to quality of life in breast cancer patients who undergo mastectomy compared to breast conservation which has been shown have lower quality of life after mastectomy in the overall breast cancer patient population.^15^

Among patients undergoing GACMS, multiple surgical options exist including free nipple grafts, double incision mastectomy, wise pattern reduction, nipple-sparing GACMS, and keyhole incision.^16^ Acknowledging the heterogeneity within SGM patients undergoing definitive oncologic surgery for breast cancer treatment requires a comprehensive surgical discussion that includes surgical options that align with an individual’s sexual orientation and/or gender identity. Utilization of patient-reported outcome data for gender-affirming surgeries such as the recently developed GENDER-Q may aid in measuring patient surgical satisfaction for gender minority patients after breast cancer treatment.^17^ Presenting all surgical options to patients that align with their gender and their desired cosmetic outcome while simultaneously ensuring an appropriate oncologic resection is key to optimizing both patient satisfaction and oncologic outcomes.

A suggested framework to ensure a comprehensive surgical discussion that incorporates sexual orientation and/or gender identity entails open-ended questions and use of inclusive language to counsel patients and understand surgical goals to best aid with patient-centered decision making. A review of best practices for gender-affirming treatment for nonbinary and transgender patients include gender neutral questionnaires, inclusive nongendered support groups, cultural humility training for staff, affirming imagery, gender neutral bathrooms and facilities and medical record documentation inclusive of affirmed name, pronouns and organ inventory.^18^ Creating safe spaces in breast oncology for SGM patients may help in decreasing the marginalization that SGM breast cancer respondents experienced while navigating oncologic treatment.

The authors would recommend providing focused educational initiatives to improve interactions and care for SGM patients with breast cancer. Opportunities include comprehensive provider training on sexual orientation and gender identity and implicit bias, developing best practices for caring for SGM individuals, and establishing toolkits to facilitate shared decision-making with SGM patients and their multidisciplinary breast cancer team. Findings from this research study demonstrate the need for future research efforts regarding SGM patients and breast oncologic care. Intersectional identities have been shown to have worsened delays in oncologic care^8^ and are a notable direction for future SGM cancer research. The current sociopolitical landscape has the potential to worsen outcomes of previously marginalized groups in seeking healthcare and disclosure of one’s sexual orientation and/or gender identity, especially in locations where anti-discriminatory legislation and laws are not prevalent. Standardization of SOGI data collection in health systems while maintaining patient confidentiality may improve patient navigation through oncologic care. Standardization could include adding organ inventory for nonbinary, transgender or gender diverse patients as well as sexual orientation and gender identity and pronouns to intake forms and research questionnaires.

Limitations include those inherent to survey-based research including respondent selection bias, low response rates, and the inability to determine entire population size that had the opportunity to take the survey. Breast cancer subtype was not captured, nor were granular details regarding treatment specifics, including omitting selected therapies. International respondents noted that surgical choice was not possible in their treating countries, thus not all the surgical decisions are driven by patient driven selection as 19% (n = 5) respondents had surgery performed outside the United States. Additionally, the SGM community represents a heterogeneous spectrum group of individuals with distinct preferences, thus limiting generalizability; however, the findings presented here underscore improvement opportunities to serve SGM individuals faced with a breast cancer diagnosis.

## 5. Conclusion

In this survey study of SGM respondents with a prior breast cancer diagnosis, we identified that SGM identity is relevant to breast cancer treatment choices, especially regarding surgical decision-making. Furthermore, many respondents reported misgendering and discrimination which demonstrates the need for comprehensive provider training and strategic initiatives within healthcare systems to provide sensitive and inclusive patient care. Surgeons should collect and incorporate patient sexual orientation and gender identity to ensure a shared patient-centered approach in the surgical decision-making process for SGM individuals with breast cancer.

## Supplementary Material

S2

S1

## Figures and Tables

**Table 1:** Cohort characteristics of sexual and gender minority respondents with breast cancer.

Response	N^a^ = 31 (%)

Gender Identity^b^	

Cisgender woman	24 (77%)
Nonbinary	5 (16%)
Transgender man	1 (3%)
Unknown	1 (3%)

Sexual Identity^b^	

Asexual	1 (3%)
Bisexual	7 (23%)
Heterosexual/straight	1 (3%)
Lesbian/gay/homosexual	18 (58%)
Pansexual	4 (13%)
Queer	4 (13%)
Other	1 (3%)

Sex Assigned at Birth	

Female	31 (100%)

Race and Ethnicity	

Black/African American	1 (3%)
White/Caucasian	27 (87%)
Unknown	3 (10%)

Insurance	

Private	17 (55%)
Medicare	3 (10%)
Medicaid	4 (13%)
Other government insurance	3 (10%)
None	1 (3%)
Unknown	3 (10%)

Education	

High School/Graduate Equivalency Degree	1 (3%)
Some College	5 (16%)
Associate Degree	1 (3%)
Bachelor’s degree	9 (29%)
Graduate Degree	12 (39%)
Unknown	3 (10%)

Geographic Location for Surgery	

Northeast United States	9 (29%)
Southern United States	2 (6%)
Midwest United States	9 (29%)
Western United States	2 (6%)
Outside United States	5 (16%)
	
Unknown	4 (13%)
Age at Breast Cancer Diagnosis, years	

≤35	6 (19%)
36–50	14 (45%)
51–75	11 (35%)

Cancer Stage at Diagnosis	

0	4 (13%)
I-III	27 (87%)

Method of Breast Cancer Detection^b^	

Screening Mammogram	15 (48%)
Patient Palpated	14 (45%)
Healthcare Exam	2 (6%)
Symptom (pain, nipple discharge)	4 (13%)

Genetic Testing	

Yes, results prior to surgery	16 (52%)
Yes, results after surgery	5 (16%)
Yes, tested after surgery	3 (10%)
No	4 (13%)

Unknown	3 (10%)

Pathogenic Germline Variant (n = 24)	
Yes	4/24 (17%)
No	20/24 (83%)

Surgery for Breast Cancer (n = 31)	

Yes	28 (90%)
No	3 (10%)

Surgical Management^b^ (n = 28)	

Unilateral Mastectomy	5/28 (18%)
Not Nipple Sparing	1/3
Nipple Sparing	2/3
Bilateral Mastectomy (± nipple sparing)	12/28 (43%)
Not Nipple Sparing	12/12
Nipple Sparing	0
Lumpectomy	14/28 (50%)

Reconstructive Surgery (n = 28)	

Immediate Reconstruction	4/28 (14%)
Delayed Reconstruction	4/28 (14%)
Flat Aesthetic closure	5/28 (18%)
No reconstruction	15/28 (54%)

Reconstructive Surgery Type	

Flat closure	5/28 (18%)
Tissue Expander to Implant	1/28 (4%)
Direct To Implant	3/28 (11%)
Autologous Flap	2/28 (7%)
Other (Goldilocks, reduction)	2/28 (7%)
No reconstruction	15/28 (54%)

Surgical Recommendation (n = 28)	

Unilateral Mastectomy	4/28 (14%)
Bilateral Mastectomy	5/28 (18%)
Lumpectomy	10/28 (36%)
Other	9/28 (32%)

Had Choice in Surgery (n = 28)	

Yes	23/28 (82%)
No	5/28 (18%)

Gender Identity and/or Sexual Orientation Had Impact on Surgical Choice (n = 28)	

Yes	13/28 (46%)
No	15/28 (54%)

Disclosed Sexual Orientation or Gender Identity to Breast Cancer Surgeon^b^	

Yes	12 (39%)
No, was not asked	8 (26%)
No, did not think would factor into treatment	3 (10%)
No, fear of marginalization/discrimination	2 (6%)
Other	8 (26%)

Experienced discrimination and/or marginalization during treatment	

Yes	9 (29%)
No	19 (62%)
Unknown	3 (10%)

Comfort with Appearance/Feel of Chest/Breast Prior to Surgery	

Very Uncomfortable	5 (16%)
Somewhat Uncomfortable	6 (19%)
Neutral	3 (10%)
Somewhat Comfortable	2 (6%)
Very Comfortable	13 (42%)
Unknown	2 (6%)

Comfort with Appearance/Feel of Chest/Breast After Surgery (n = 28)	

Very Uncomfortable	3/28 (11%)
Somewhat Uncomfortable	3/28 (11%)
Neutral	0 (0%)
Somewhat Comfortable	13/28 (46%)
Very Comfortable	8/28 (39%)

Unknown	1/28 (4%)

Gender Affirming Surgery Prior to Diagnosis	

Yes	1 (3%)
No	19 (61%)
Unknown	11 (35%)

Gender Affirming Hormone Therapy	

Yes, 1–5 years prior to diagnosis	2 (6%)
Yes, ≥10 years prior to diagnosis	1 (3%)
No, never taken	25 (81%)
Unknown	3 (10%)

Radiation Therapy	

Yes	15 (48%)
No	11 (35%)
History of radiation (not for this diagnosis)	1 (3%)
Unknown	4 (13%)

Chemotherapy	

Neoadjuvant	3 (13%)
Adjuvant	5 (16%)
History of chemotherapy (not for this diagnosis)	1 (3%)
No	17 (55%)
Unknown	5 (16%)

Endocrine Therapy	

Yes	14 (45%)
No	13 (42%)
Unknown	4 (13%)

a Of note, survey questions were optional so not all respondents answered each question.

b Multiple responses allowed; total may add up >100%.

**Table 2:** Qualitative analysis of free text responses in sexual and gender minority respondents with breast cancer (SOGI: Sexual Orientation and Gender Identity).

Category/Code	Participant Quote

*Surgical Decision-Making*	
Surgeon Driven	“Surgeon recommended double mastectomy”
Patient Driven	“I knew I wanted a double mastectomy”
Shared Decision	“[Surgeon] presented options, but I made the choice”
*SOGI Identity Influence on Surgery*	
Gender Influenced	“[I’m] nonbinary and always considered top surgery”
Sexual orientation influenced	“It made the choice to go flat easier”
Other Factors	“... my hope in being a mother and breastfeeding”
*Disclosure of SOGI Data*	
Felt safe/comfortable	“Felt safe to include [the surgeon]”
*Experience of Marginalization During Treatment*	
Misgendering	“Receptionist misgendered me as a man, but it happens all the time”
Overt discrimination	“I was deemed worthy of a psych evaluation ...”
Judgment from cultural norms	“Surgeon was horrified I did not want reconstruction”
Post-Operative Concerns	
Cosmetic dissatisfaction	“Concaveness is jarring”
Persistent gender dysphoria	“[My breasts] give me the feeling that I am perceived as a woman”

## Appendix A. Supplementary data

Supplementary data to this article can be found online at https://doi.org/10.1016/j.amjsurg.2026.116947.
