# Supplementary material for: Experiences and challenges of sexual and gender minority patients undergoing breast cancer treatment
Source: Am J Surg. Author manuscript; Available in PMC 2026 May 23. (PMC13197973; doi:10.1016/j.amjsurg.2026.116947)
Supplement: S2 [file NIHMS2167972-supplement-S2.docx]

**SUPPLEMENTAL MATERIAL**

Experiences and Challenges of Sexual and Gender Minority Patients Undergoing Breast Cancer Treatment

Appendix 1: Survey

Appendix 2: COREQ (COnsolidated criteria for REporting Qualitative research) Checklist

**Appendix 1:**

**Breast Cancer in LGTQIA+ Community Survey**

By completing this survey, you are agreeing to participate in an anonymous research study that is seeking to learn more about LGBTQIA patients who have experienced breast cancer and breast cancer treatment.

1. What is your gender identity?

- Cisgender man
- Cisgender woman
- Nonbinary
- Transgender man
- Transgender woman
- Other: _____________________

2. What is your sexual identity?

- Asexual
- Bisexual
- Heterosexual/straight
- Lesbian/Gay/homosexual
- Pansexual
- Queer
- Other:____________

3. What is your sex assigned at birth?

- Female
- Male
- Intersex
- Choose not to answer
- Other:____________

4. Have you been diagnosed with breast cancer?

- Yes
- No → (disqualified)

5. Did you have surgery for your breast cancer?

- Yes
- No

*Please answer the following questions pertaining to your breast cancer diagnosis:*

6. How old were you when you were diagnosed with breast cancer?

If you were diagnosed with breast cancer more than once, please enter your age at the time of the first diagnosis:

- 0–17 years old
- 18– 35 years old
- 36–50 years old
- 51–75 years old
- >75 years old

7. What was your cancer stage **at the time of diagnosis**?

If you were diagnosed with breast cancer more than once, please enter the stage at the time of the first diagnosis:

- 0
- 1, 2, or 3
- 4 (metastatic, spread to other organs)
- I was told my cancer was “early stage” but do not know the exact number.
- I was told my cancer was “late stage” or “advanced” but do not know the exact number.
- I do not know or I do not remember.

8. How was your breast cancer detected?

- Routine mammogram (screening test)
- I felt it
- Healthcare provider detected it
- Symptom: (such as breast/chest pain, nipple discharge)
- Other:__________

The following questions refer to your breast cancer surgery and treatment:

9. What operation did you have? Check all that apply

**Mastectomy** is complete removal of the breast

**Lumpectomy** is removal of the tumor only; the breast is not removed

- Single (only side of the cancer) mastectomy and removal of the nipple
- Single (only side of the cancer) mastectomy without removal of the nipple
- Double (both sides) mastectomy and removal of one or both nipples
- Double (both sides) mastectomy without removal of either nipple
- Lumpectomy and removal of the nipple
- Lumpectomy without removal of the nipple

10. Did you have any type of reconstructive surgery?

**Reconstructive surgery** might be performed by your breast surgeon or a plastic surgeon and could include procedures to fill in a defect after lumpectomy, make the chest wall after mastectomy more natural-appearing, or re-create a nipple if the nipple was removed.

- I had reconstructive surgery at the time of lumpectomy or mastectomy.
- I had reconstructive surgery after the lumpectomy or mastectomy.
- I chose to have an aesthetic flat closure after mastectomy.*
- I did not have any reconstructive surgery → (skip to Q13).

11. What type of reconstruction did you have? Please read the choices carefully and choose the one that best fits your situation:

- Tissue expander with eventual implant placement or plan for implant placement
- Direct to implant (no tissue expander)
- Flap using your own body fat and/or muscle including DIEP, TRAM, latissimus flap and other
- Combination of tissue expander/implant + flap
- Liposuction and fat injections
- Use of skin and fat (from chest area)
- Other, please specify:______

12. Which of the following best characterizes **the discussion with your surgeon** regarding options for surgery?

- My surgeon recommended single mastectomy.
- My surgeon recommended double mastectomy.
- My surgeon recommended lumpectomy.
- Other, please specify:______

13. Do you feel you had a choice in the decision for surgery?

- Yes
- No

14. Do you feel you had adequate information about all of your surgical options so that you could make the right decision for you?

- Yes
- No

15. Did your gender identity or sexual orientation have an impact on your surgical choice?

- Yes
  - If yes, description of impact____________________
- No

16. Did you disclose your sexual orientation or gender identity to your breast cancer surgeon?

- Yes – if yes continue to question 19
- No

17. If you did not disclose your sexual orientation or gender identity to your provider, why was that?

- Was not asked
- Did not think it would factor into surgery, breast cancer treatment
- Fear of discrimination/marginalization
- Other________________

18. During your cancer treatment, did you ever experience discrimination or marginalization related to your gender identity or sexual orientation?

- Yes- Free response if you feel comfortable providing details
- No

19. How comfortable were you with the appearance and feel of your chest and/or before surgery?

- Very comfortable, the appearance or feel do not bother me
- Somewhat comfortable, the appearance or feel do bother me somewhat
- Neutral–neither comfortable nor uncomfortable
- Somewhat uncomfortable: I am somewhat bothered by the appearance or feel.
- Very uncomfortable: I am very bothered by the appearance or feel.

20. How comfortable are you with the appearance and feel of your chest and/or breast and scar after surgery?

- Very comfortable, the appearance or feel do not bother me
- Somewhat comfortable, the appearance or feel do bother me somewhat
- Neutral–neither comfortable nor uncomfortable
- Somewhat uncomfortable: I am somewhat bothered by the appearance or feel.
- Very uncomfortable: I am very bothered by the appearance or feel.

21. Have you had gender affirming top surgery (gender affirming mastectomy, gender affirming augmentation)?

- Yes
- No- if no proceed to question 25*

22. Did you have top surgery (gender affirming chest/breast surgery) before your diagnosis of breast cancer?

- Yes- had top surgery prior to diagnosis
- No- did not have top surgery before diagnosis
- No- I have never had a gender affirming top surgery

23. Please comment what bothers you most about the appearance or feel of your chest and scar area: (free response)

24. Have you had radiation therapy to the breast or chest, **on the side of mastectomy**? Please read the choices carefully and choose the one that best fits your situation:

- I had radiation **before** my mastectomy **for previous breast cancer**.
- I had radiation **after** my mastectomy **due to breast cancer**.
- I had radiation for Hodgkin’s lymphoma or other medical condition **before my mastectomy**.
- I had radiation for Hodgkin’s lymphoma or other medical condition **after my mastectomy**.
- I received radiation but did not or have not yet had surgery.
- I have not had radiation to the breast or chest.

25. Did you receive **chemotherapy**? Please **do not consider endocrine therapy** such as tamoxifen or aromatase inhibitors (anastrozole/arimidex, letrozole/femara, exemestane/aromasin) in answering this question. Please read the choices carefully and choose the one that best fits your situation:

- I received chemotherapy **before** my mastectomy surgery, **for previous breast or non-breast cancer**.
- I received chemotherapy **before** my mastectomy surgery, for **my breast cancer**.
- I received chemotherapy **after** my mastectomy surgery, for **my breast cancer**.
- I received chemotherapy **after** my mastectomy surgery, for **non-breast** cancer.
- I received chemotherapy but did not or have not yet had surgery.
- I have not received chemotherapy.

26. Did you receive **endocrine therapy** (anastrozole/arimidex, letrozole/femara, exemestane/aromasin) as part of your breast cancer treatment?

- I have not received endocrine therapy for my breast cancer
- I received endocrine therapy after my breast cancer surgery
- I received endocrine therapy prior to my breast cancer surgery

27. Have you ever taken **gender affirming hormone therapy**?

- Yes- for 1-5 years prior to diagnosis
- Yes- for 6-10 years prior to diagnosis
- Yes- for 11-20 years prior to diagnosis
- No- I have never taken gender affirming hormone therapy

*Please answer the following questions pertaining to your family history and genetics:*

28. Did you have genetic testing before surgery or after surgery?

- I was tested before surgery and got my results before surgery.
- I was tested before surgery but didn’t get my results until after surgery.
- I was tested after surgery.
- I was tested but I did not or have not yet had surgery.
- I did not undergo genetic testing → (skip to Q34) .

29. Did you undergo genetic testing for a BRCA (BRCA 1 or 2) or other genetic mutation?

- I was tested for BRCA 1 and 2.
- I was tested for BRCA 1 and 2 as well as other breast cancer related genes.

30. Were you found to have an abnormal BRCA gene (BRCA 1 or 2 mutation) or other abnormal gene that might contribute to breast cancer risk? **Please consider a variant of uncertain significance (VUS) to be “no” when responding to this question:**

- Yes
- No

*The following questions pertain to your current demographic information:*

31. In what region was your surgery performed?

- Northeast US
- Southern US
- Midwest US
- West US
- Outside the US

32. What best describes your racial / ethnic background? Please check all that apply:

- American Indian or Alaska native
- Asian
- Black or African American
- Hispanic, Latino or Spanish origin
- Native Hawaiian or Pacific Islander
- White
- Other, please specify

33. What type of insurance did you have at the time of your surgery?

- Private insurance–individual policy, not from employer
- Private insurance–group policy from employer
- Medicare with or without a secondary insurance
- Medicaid
- Other government insurance
- None
- don’t know or I don’t remember

34. Is there any other information you would like the research team to know about you and your surgery? (optional-write in)

Appendix 2. **COREQ (COnsolidated criteria for REporting Qualitative research) Checklist**
